# Retraction: Silencing Alpha-Fetoprotein Inhibits VEGF and MMP-2/9 Production in Human Hepatocellular Carcinoma Cell

**DOI:** 10.1371/journal.pone.0244942

**Published:** 2020-12-31

**Authors:** The PLOS ONE Editors

Following the publication of this article [1], concerns were raised regarding the results presented in Fig 3 and Fig 4. Specifically,

- The β-actin panel of Fig 3Ca appears similar to the β-actin panel of Fig 4A when horizontally compressed and vertically stretched. The authors have explained that the same experimental controls were used for these figures.
- Multiple unusually repetitive clusters have been detected within Fig 4B, Fig 4C, and Fig 4D. In addition, in some areas of Fig 4B the background signal does not appear to match the background signal of the immediate surrounding area, and in some areas this background signal appears to cut off other signals at the border of the mismatching areas.

The authors provided underlying data for the panels B, C, and D presented in Fig 4, and explained that the similarities between the dots are the result of the non-randomised nature of the Transwell plate micro-pores. However, the underlying data provided were not an exact match with the published panels and they did not resolve the concerns pertaining to the repetition detected in these panels.

In light of the concerns affecting multiple figure panels of Fig 4 that question the integrity of these data, the *PLOS ONE* Editors retract this article.

WM, ZL, ZB, YaL, JY, TL, JY, WZ, KZ, and YuL did not agree with the retraction. WM, ZB, JY, TL, JY, WZ, KZ, and YuL stand by the article’s findings. HZ either did not respond directly or could not be reached.
